# Predicting Postoperative Mortality With Deep Neural Networks and Natural Language Processing: Model Development and Validation

**DOI:** 10.2196/38241

**Published:** 2022-05-10

**Authors:** Pei-Fu Chen, Lichin Chen, Yow-Kuan Lin, Guo-Hung Li, Feipei Lai, Cheng-Wei Lu, Chi-Yu Yang, Kuan-Chih Chen, Tzu-Yu Lin

**Affiliations:** 1 Graduate Institute of Biomedical Electronics and Bioinformatics National Taiwan University Taipei Taiwan; 2 Department of Anesthesiology Far Eastern Memorial Hospital New Taipei City Taiwan; 3 Research Center for Information Technology Innovation Academia Sinica Taipei Taiwan; 4 Department of Computer Science Columbia University New York, NY United States; 5 Department of Computer Science and Information Engineering National Taiwan University Taipei Taiwan; 6 Department of Electrical Engineering National Taiwan University Taipei Taiwan; 7 Department of Mechanical Engineering Yuan Ze University Taoyuan Taiwan; 8 Department of Information Technology Far Eastern Memorial Hospital New Taipei City Taiwan; 9 Section of Cardiovascular Medicine Cardiovascular Center Far Eastern Memorial Hospital New Taipei City Taiwan; 10 Department of Internal Medicine Far Eastern Memorial Hospital New Taipei City Taiwan

**Keywords:** bidirectional encoder representations from transformers, deep neural network, natural language processing, postoperative mortality prediction, unstructured text, machine learning, preoperative medicine, anesthesia, prediction model, anesthesiologist, deep learning model, electronic health record, neural network

## Abstract

**Background:**

Machine learning (ML) achieves better predictions of postoperative mortality than previous prediction tools. Free-text descriptions of the preoperative diagnosis and the planned procedure are available preoperatively. Because reading these descriptions helps anesthesiologists evaluate the risk of the surgery, we hypothesized that deep learning (DL) models with unstructured text could improve postoperative mortality prediction. However, it is challenging to extract meaningful concept embeddings from this unstructured clinical text.

**Objective:**

This study aims to develop a fusion DL model containing structured and unstructured features to predict the in-hospital 30-day postoperative mortality before surgery. ML models for predicting postoperative mortality using preoperative data with or without free clinical text were assessed.

**Methods:**

We retrospectively collected preoperative anesthesia assessments, surgical information, and discharge summaries of patients undergoing general and neuraxial anesthesia from electronic health records (EHRs) from 2016 to 2020. We first compared the deep neural network (DNN) with other models using the same input features to demonstrate effectiveness. Then, we combined the DNN model with bidirectional encoder representations from transformers (BERT) to extract information from clinical texts. The effects of adding text information on the model performance were compared using the area under the receiver operating characteristic curve (AUROC) and the area under the precision-recall curve (AUPRC). Statistical significance was evaluated using *P*<.05.

**Results:**

The final cohort contained 121,313 patients who underwent surgeries. A total of 1562 (1.29%) patients died within 30 days of surgery. Our BERT-DNN model achieved the highest AUROC (0.964, 95% CI 0.961-0.967) and AUPRC (0.336, 95% CI 0.276-0.402). The AUROC of the BERT-DNN was significantly higher compared to logistic regression (AUROC=0.952, 95% CI 0.949-0.955) and the American Society of Anesthesiologist Physical Status (ASAPS AUROC=0.892, 95% CI 0.887-0.896) but not significantly higher compared to the DNN (AUROC=0.959, 95% CI 0.956-0.962) and the random forest (AUROC=0.961, 95% CI 0.958-0.964). The AUPRC of the BERT-DNN was significantly higher compared to the DNN (AUPRC=0.319, 95% CI 0.260-0.384), the random forest (AUPRC=0.296, 95% CI 0.239-0.360), logistic regression (AUPRC=0.276, 95% CI 0.220-0.339), and the ASAPS (AUPRC=0.149, 95% CI 0.107-0.203).

**Conclusions:**

Our BERT-DNN model has an AUPRC significantly higher compared to previously proposed models using no text and an AUROC significantly higher compared to logistic regression and the ASAPS. This technique helps identify patients with higher risk from the surgical description text in EHRs.

## Introduction

The prevalence of postoperative mortality is 0.5%-2.8 % in patients undergoing elective surgery [1]. The risks are attributable to the patient’s condition and can be modulated with adequate evaluation and planning during surgery and anesthesia. Several tools have been developed to predict postoperative mortality, including the American College of Surgeons’ (ACS) National Surgical Quality Improvement Program (NSQIP) risk calculator, the American Society of Anesthesiologist Physical Status (ASAPS), the risk quantification index, the risk stratification index, and the preoperative score [2-5]. Although these classification systems consider the patient’s general condition and surgery category, preoperative vital signs and laboratory data—which are critical in predicting postoperative mortality—are not typically included [6]. Moreover, a patient’s surgical information is commonly written as text in the medical record. Although reading this information helps anesthesiologists evaluate the risk of the surgery, it is difficult to include it in a classification tool. These deficiencies make it challenging to identify the small groups of patients with higher risks. Better tools for predicting postoperative mortality remain under investigation.

Machine learning (ML) is widely applied to medical problems, including for predicting postoperative mortality [6-11]. ML models can automatically predict postoperative mortality using electronic health records (EHRs) before surgery, and they achieve a superior area under the receiver operating characteristic curve (AUROC) than previous methods [6]. To stratify surgery types, previous studies have used the Current Procedural Terminology (CPT) codes or *International Classification of Diseases* (ICD) codes for surgical information [2,6,7,9,12]. These methods are not widely applicable, because the CPT is not implemented worldwide and ICD codes are seldom recorded before surgery. In addition, because this surgical information is written in the medical record by surgeons before surgery, using this text in models may improve the prediction of postoperative mortality.

Compared to structured EHRs, unstructured clinical text requires meaningful concept embeddings to be extracted before model training, making it more challenging [13]. However, including this unstructured text improves the advanced prediction of unfavorable clinical outcomes [14-16]. Bidirectional encoder representations from transformers (BERT) is a contextualized embedding method that preserves the distance of meanings with multihead attention [17]. After pretrained on the relevant corpora and proper architecture modification, BERT extracts meaningful embeddings from clinical text [18,19].

This study aims to develop a model to predict 30-day postoperative mortality before surgery that performs better than state-of-the-art models. Our contribution is including free (ie, unstructured) text in postoperative mortality prediction by proposing a deep neural network (DNN) model with BERT. We investigate the effectiveness of unstructured clinical texts (eg, preoperative diagnosis and proposed procedures) in predicting postoperative mortality.

## Methods

### Data Extraction

**Figure 1:**
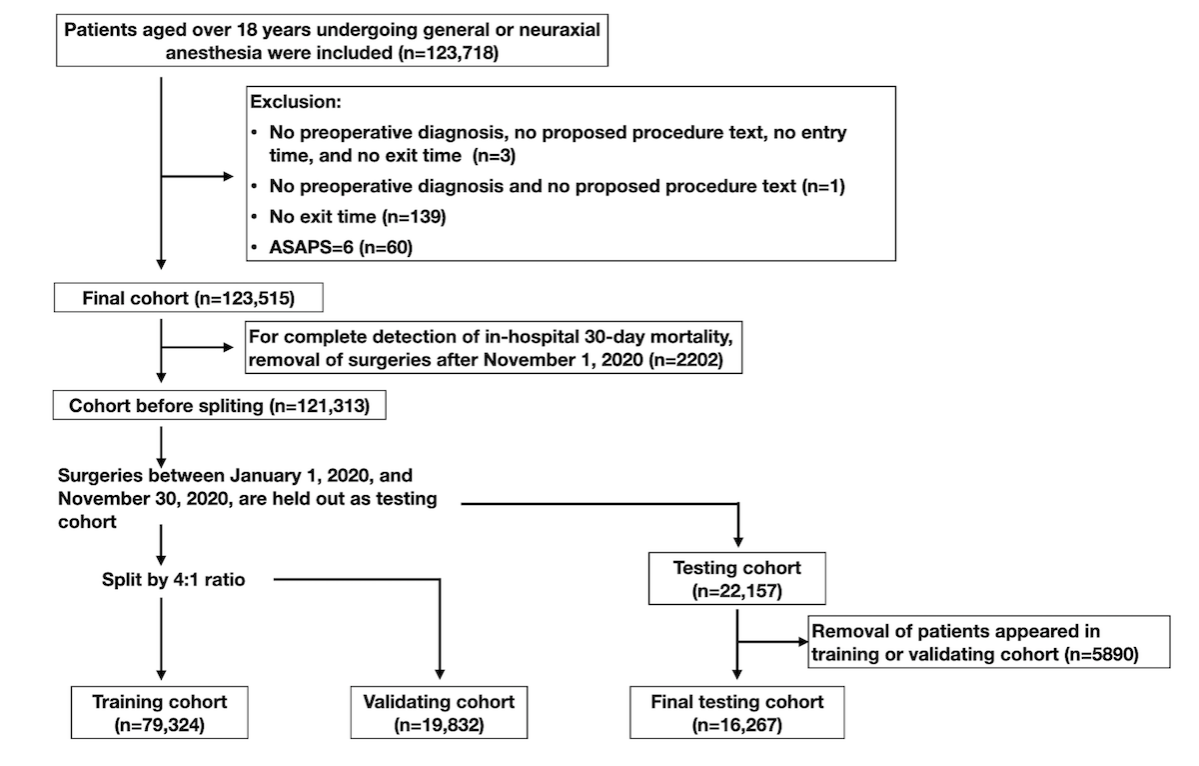
Flow diagram. ASAPS: American Society of Anesthesiologist Physical Status.

This study aims to predict in-hospital 30-day postoperative mortality using preoperative anesthesia assessments. Data were collected from the electronic health system of the Far Eastern Memorial Hospital, a large academic medical center in Taiwan. Preoperative anesthesia assessment records and discharge summaries were included. Overall, 5 years’ worth of retrospective data were collected from January 1, 2016, to December 30, 2020. The last version of the anesthesia assessment was included for each surgery. Patients over 18 years of age who underwent at least 1 surgical procedure under general or neuraxial anesthesia were included. Cases with an ASAPS of 6 were excluded. Records lacking entry time, exit time, preoperative diagnosis, or proposed procedure text were excluded. The in-hospital 30-day postoperative mortality was defined by a discharged route of “expired” and “critical against-advice discharge” (when the patient wants to die at home) without future admission. Discharges within 30 days after surgery were identified and labeled as “true”; those occurring outside this window were marked as “false.” The end date of the testing set was November 30, 2020, 30 days before the end of the collected data, to ensure complete 30-day mortality detection (Figure 1).

### Ethical Approval

The Institutional Review Board of the Far Eastern Memorial Hospital approved this retrospective study and waived the requirement of informed consent (#109129-F and #110028-F).

### Data Description

We collected 123,718 surgery results for patients aged over 18 years. After applying the exclusion criteria, a cohort of 123,515 (99.8%) patients who underwent surgeries remained. A final cohort of 121,313 (98.2%) patients was used after removing those who underwent surgeries after November 30, 2020 (Figure 1). The training, validation, and testing cohorts finally contained 79,324 (68.7%), 19,832 (17.2%), and 16,267 (14.1%) of 115,423 patients. Patient characteristics of the training, validation, and testing cohorts are listed in Table 1. In the overall cohort, most patients had an ASAPS of 2 or 3. Overall, 107,176 (88.5%) of patients were under general anesthesia. The most prevalent comorbidities were hypertension (n=43,391, 35.8%), followed by diabetes (n=24,314, 20.0%). A total of 1562 (1.3%), 997 (1.3%), 249 (1.3%), and 215 (1.3%) patients died within 30 days of surgery in the overall, training, validation, and testing cohorts, respectively. Multimedia Appendix 1 present a summary of the laboratory data and preoperative vital signs.

**Table 1:** Characteristics of the cohort. Categorical variables are represented as frequency (%). Continuous variables are represented as the median (25th, 75th percentile). The testing cohort was split by time between the training and validation cohorts, and those cases arising from the training and validation cohorts were removed to prevent data leakage (n=5890, 4.9%).

Feature			Training cohort (N=79,324)		Validation cohort (N=19,832)		Testing cohort (N=16,267)		Overall cohort (N=121,313)
Age (years), median (25th, 75th percentile)			54 (40, 66)		54 (40, 66)		53 (39, 65)		55 (41, 66)
Male sex, n (%)			40,444 (51.0)		9922 (50.0)		8101 (49.8)		61,485 (50.7)
Height (cm), median (25th, 75th percentile)			162 (157, 168)		162 (156, 168)		162 (157, 169)		162 (157, 168)
Weight (kg), median (25th, 75th percentile)			64 (56, 74)		64 (56, 74)		65 (56, 75)		64 (56, 74)
BMI, median (25th, 75th percentile)			24 (22, 27)		24 (22, 27)		24 (22, 27)		24 (22, 27)
**ASAPS^a^, n (%)**									
	1	2925 (3.7)		739 (3.7)		660 (4.1)		4404 (3.6)	
	2	54,056 (68.15)		13,549 (68.3)		11,508 (70.7)		82,588 (68.1)	
	3	20,842 (26.3)		5155 (26.0)		,654 (22.5)		31,878 (26.3)	
	4	1345 (1.70)		355 (1.8)		397 (2.4)		2204 (1.8)	
	5	156 (0.2)		34 (0.2)		48 (0.3)		239 (0.2)	
ASA^b^ emergency, n (%)			6379 (8.0)		1615 (8.1)		1678 (10.3)		9942 (8.2)
**Anesthesia type, n (%)**									
	General	69,898 (88.3)		17,497 (88.4)		14,486 (89.2)		107,176 (88.5)	
	Neuraxial	9297 (11.7)		2303 (11.6)		1748 (10.8)		13,929 (11.5)	
**Emergency level of surgery, n (%)**									
	Elective	62,226 (78.5)		15,455 (77.9)		12,000 (73.8)		94,816 (78.2)	
	Urgent	13,800 (17.4)		3,567 (18.0)		3,356 (20.6)		21,342 (17.6)	
	Emergency	2849 (3.6)		708 (3.6)		801 (4.9)		4484 (3.7)	
	Immediate	449 (0.57)		102 (0.51)		110 (0.7)		671 (0.6)	
**Preoperative location, n (%)**									
	Ward	47,187 (59.5)		11,788 (59.4)		9824 (60.4)		72,045 (59.4)	
	Outpatient	18,386 (23.2)		4463 (22.5)		2995 (18.4)		27,830 (22.9)	
	Emergency department	10,083 (12.7)		2592 (13.1)		2283 (14.0)		15,247 (12.6)	
	Intensive care unit	3668 (4.6)		989 (5.0)		1165 (7.2)		6191 (5.1)	
**Surgery department, n (%)**									
	Urology	14,760 (18.6)		3630 (18.3)		2665 (16.4)		22,471 (18.5)	
	General	11,416 (14.4)		2926 (14.8)		2457 (15.1)		17,608 (14.5)	
	Orthopedics	10,976 (13.8)		2748 (13.9)		2338 (14.4)		16,772 (13.8)	
	Gynecology^c^	10,206 (12.9)		2,578 (13.0)		2,302 (14.2)		15,679 (12.9)	
	Cardiovascular	8692 (11.0)		2086 (10.5)		1491 (9.2)		13,049 (10.8)	
	Otolaryngology	6193 (7.8)		1505 (7.6)		1223 (7.5)		9427 (7.8)	
	Plastic surgery	5116 (6.5)		1294 (6.5)		1077 (6.6)		7821 (6.4)	
	Neurosurgery	3233 (4.1)		833 (4.2)		727 (4.5)		4955 (4.1)	
	Traumatology	2808 (3.5)		740 (3.7)		722 (4.4)		4357 (3.6)	
	Thoracic surgery	2006 (2.5)		514 (2.6)		430 (2.6)		3104 (2.6)	
	Colorectal surgery	1679 (2.1)		423 (2.1)		331 (2.0)		2574 (2.1)	
	Others	2239 (2.8)		555 (2.8)		504 (3.1)		3496 (2.9)	
**Comorbidity, n (%)**									
	Diabetes mellitus	15,906 (20.1)		3863 (19.5)		2812 (17.3)		24,314 (20.0)	
	Hyperlipidemia	8704 (11.0)		2119 (10.7)		1740 (10.7)		13,678 (11.3)	
	Hypertension	28,462 (35.9)		7055 (35.6)		4999 (30.7)		43,391 (35.8)	
	Prior cerebrovascular accident	4355 (5.5)		1028 (5.2)		717 (4.4)		6564 (5.4)	
	Cardiac disease	13,215 (16.7)		3254 (16.4)		2227 (13.7)		20,156 (16.6)	
	Chronic obstructive pulmonary disease	1549 (2.0)		380 (1.9)		286 (1.8)		2428 (2.0)	
	Asthma	3024 (3.8)		762 (3.8)		592 (3.6)		4626 (3.8)	
	Hepatic disease	9118 (11.5)		2299 (11.6)		1664 (10.2)		13,887 (11.4)	
	Renal disease	12,471 (15.7)		3095 (15.6)		1466 (9.0)		18,874 (15.6)	
	Bleeding disorder	11,243 (14.2)		2684 (13.5)		2122 (13.0)		17,543 (14.5)	
	Prior major operations	54,356 (68.5)		13,592 (68.5)		10,040 (61.7)		83,490 (68.8)	
	Smoking	20,235 (25.5)		5098 (25.7)		3719 (22.9)		30,433 (25.1)	
	Drug allergy	11,662 (14.7)		2959 (14.9)		2190 (13.5)		18,092 (14.9)	
	Consciousness	69,858 (88.1)		17,461 (88.0)		15,107 (92.9)		107,906 (88.9)	
30-day mortality, n (%)			997 (1.3)		249 (1.3)		215 (1.3)		1562 (1.3)

^a^ASAPS: American Society of Anesthesiologist Physical Status.

^b^ASA: American Society of Anesthesiologists.

^c^The gynecology department consists of gynecology and obstetrics.

### Data Preparation

The input features included patient characteristics (age, height, weight, BMI, sex, ASAPS, ASA emergency status, department, preoperative location, and anesthesia type), surgery characteristics (emergency level, preoperative diagnosis, and proposed procedure), comorbidities (diabetes mellitus, hyperlipidemia, hypertension, cerebrovascular accident, cardiac disease, chronic obstructive pulmonary disease, asthma, hepatic disease, renal disease, bleeding disorder, major operations, smoking, and drug allergy), preoperative laboratory data (hemoglobin, platelet, international normalized ratio, prothrombin time, activated partial thromboplastin time, creatinine, aspartate transaminase, alanine transaminase, blood sugar, serum sodium, and serum potassium), and preoperative vital signs (body temperature, oxygen saturation, heart rate, respiratory rate, systolic and diastolic blood pressure, and consciousness status); see Table 2.

Continuous features (eg, age, height, weight, latest laboratory data before surgery, and preoperative vital signs) were standardized by subtracting the mean and scaling to variance. Outliers were regarded as input errors and treated as missing data. Multimedia Appendix 2 lists the definitions of the outliers. Missing values were imputed with the median value of the data set for continuous features.

Categorical features with only 2 classes (eg, sex, comorbidities, ASA emergency status, and consciousness status) were converted into binary encoding. All other categorical features (eg, ASAPS [5 classes], department [22 classes], emergency level [4 classes], preoperative location [4 classes], and anesthesia type [4 classes]) were transformed into one-hot encodings. Missing data were imputed with the majority category of the training data set. The preoperative diagnoses and proposed procedures were expressed as free text. Characters other than alphabetical and numerical ones were removed (eg, Chinese characters [typically notes for colleagues only] and punctuation). English stop words providing no helpful information to the model (eg, “a,” “in,” and “the”) were removed using the Natural Language Toolkit [20].

We used the previous 4 years’ surgery results to predict the last year results. Patients who underwent surgeries between January 1, 2016, and December 31, 2019, were selected and split into training and validation sets in a 4:1 ratio; those who underwent surgeries between January 1, 2020, and November 30, 2020, were selected as the testing set (Figure 1). Patients in the training or validation set were removed from the testing set to prevent information leakage [6].

**Table 2:** Feature groups included in the models.

Feature type		Feature classes^a^
**Patient characteristics**		
	Continuous	Age, height, weight, BMI
	Categorical	Sex (2), ASAPS^b^ (5), ASA^c^ emergency (2), department (22), preoperative location (4), anesthesia type (4)
**Surgery characteristics**		
	Categorical	Emergency level (4)
	Free text	Preoperative diagnosis, proposed procedure
**Comorbid conditions**		
	Categorical	Diabetes mellitus (2), hyperlipidemia (2), hypertension (2), cerebrovascular accident (2), cardiac disease (2), chronic obstructive pulmonary disease (2), asthma (2), hepatic disease (2), renal disease (2), bleeding disorder (2), major operations (2), smoking (2), drug allergy (2)
**Preoperative laboratory values**		
	Continuous	Hemoglobin, platelet, international normalized ratio, prothrombin time, activated partial thromboplastin time, creatinine, aspartate transaminase, alanine transaminase, blood sugar, serum sodium, serum potassium
**Preoperative vital signs**		
	Continuous	Body temperature, oxygen saturation, heart rate, respiratory rate, systolic and diastolic blood pressure
	Categorical	Consciousness status (2)

^a^The number of classes is shown in parentheses.

^b^ASAPS: American Society of Anesthesiologist Physical Status.

^c^ASA: American Society of Anesthesiologists.

### Study Design

Our results were compared with state-of-the-art models, using patient preoperative vital signs and laboratory data to predict in-hospital 30-day mortality [6]. Meanwhile, to demonstrate the effect of adding preoperative diagnoses and proposed procedures to the prediction model, we added text features and compared the performances of the highest-performing models.

**Figure 2:**
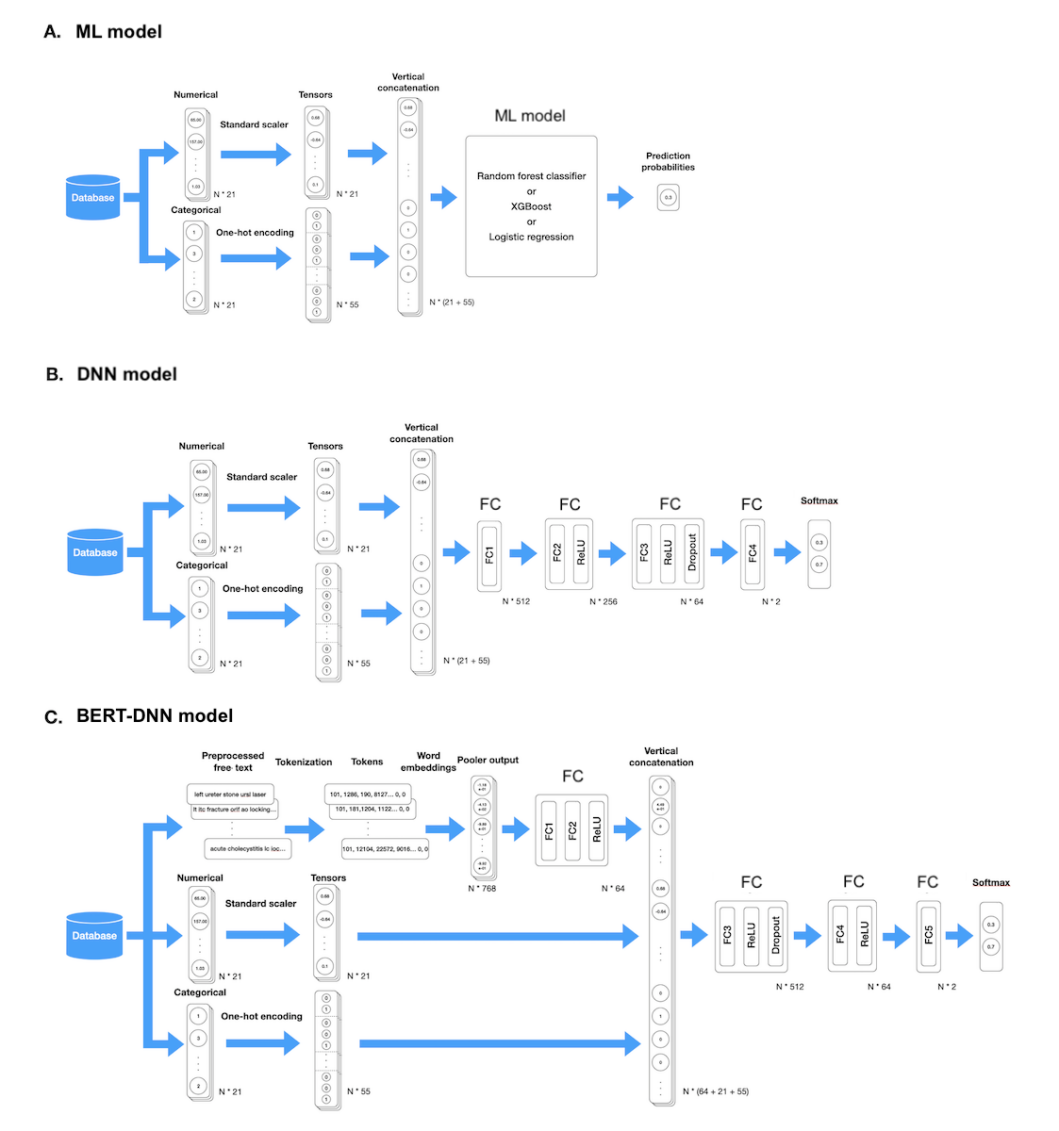
Architectures of models. BERT: bidirectional encoder representations from transformers: DNN: deep neural network; FC: fully connected; ML: machine learning; ReLU: rectified linear unit; XGBoost: extreme gradient boosting.

First, we compared the state-of-the-art models using patient and surgery characteristics (without text), comorbidities, preoperative vital signs, and laboratory data to predict the in-hospital 30-day mortality. Figure 2B shows our proposed DNN model with 4 fully connected (FC) layers and a Softmax layer output function. We compared our DNN model with other ML models, including a random forest classifier (with 2000 estimators and Gini impurity as the splitting criterion) [21], extreme gradient boosting (XGBoost, with a learning rate of 0.3 and a maximum depth of 6) [22], and logistic regression (with an L2 penalty); see Figure 2A. To balance the data while training the ML models, oversampling by 78 times was performed on the training set via the synthetic minority oversampling technique; this produced synthetic samples along a straight line between randomly selected samples in the feature space [23]. While training our DNN model, we adjusted the weight to compensate for the imbalanced classes. We added the text of preoperative diagnoses and proposed procedures to the DNN model architecture (denoted as BERT-DNN; see Figure 2C) and compared its performance with those of other models.

### Language Model and BERT-DNN Model Design

The language model extracted features from the preprocessed text. Figure 2C shows the architecture of the language model. The preprocessed texts were tokenized using the BERT tokenizer, which transformed each word fragment into a unique token designed for use in BERT’s pretraining process [17]. Then, these tokens were embedded by Bio+Clinical BERT, a variant of BERT pretrained on text from PubMed and Medical Information Mart for Intensive Care III [24]. The text information was transformed into a 768-dimension vector (the “word embeddings”) at the pooler output layer [17,24]. These word embeddings were input into 2 FC layers before concatenation with other structured features. The concatenated vectors were input into 3 FC layers and a Softmax layer output function. Figure 2C shows the architecture of the BERT-DNN model.

Cross-entropy was used as the loss function. Class weight imbalances were compensated for by setting the weights as the inverses of the different classes’ frequencies (~1:78). Further, the training data were split into training and validating sets in a 4:1 ratio to train the deep learning (DL) model. We used AdamW from the PyTorch package as the optimizer, setting a learning rate of 0.00002 for both DL models. We trained our BERT-DNN and DNN models with batch sizes of 64 and 512, respectively, until the 100th epoch. The DL model with the smallest validation loss was selected for performance comparison.

### Model Evaluation

The models were evaluated using the AUROC, the area under the precision-recall curve (AUPRC), sensitivity (also referred to as recall), specificity, precision (also called the positive predictive value), and the F1 score. The F1 score was a harmonic mean of recall and precision and was calculated as 2/[(1/recall) + (1/precision)]. Because postoperative mortalities accounted for 1.3% (1562/121,313) of our data set, classes were extremely imbalanced between the positive and negative groups. Here, the AUPRC (which calculated the average precision) was better than the AUROC for evaluating the discrimination of models [25,26]. For comparison of AUROCs, we applied a nonparametric approach proposed by DeLong et al [27] to calculate the SE of the area and the *P* value. *P*<.05 was regarded statistically significant. We calculated exact binomial 95% CIs for the AUROC. For comparison of AUPRCs, we performed bootstrapping 1000 times in the testing set to calculate the difference in areas and the 95% CI [28]. If the 95% CI for the difference in areas does not include 0, it can be concluded that these 2 areas are significantly different (*P*<.05). We performed bootstrapping 1000 times in the testing set to calculate the 95% CI for other metrics [6]. The predicted probabilities were calibrated using the histogram bins technique, using the same observed mortality in each bin of the validation set [8]. After calibration, the mean observed incidences of mortality were plotted against the mean predicted probabilities within groups in the testing set.

### Visualization of Word Embeddings

To show the correlation between increased prediction probabilities and text inputs, the *t* distributed stochastic neighbor embedding (SNE) was implemented by reducing the 768 dimensions of the language model’s pool output to 2 into a plane [29,30]. Thus, we showed the clustering of word embeddings using assorted colors for different predicted probabilities and different icons for observed mortalities. We randomly resampled 10,000 and 5000 patients who underwent surgeries in the training and testing sets, respectively, to construct this visualization. The language-model-predicted probabilities and observed mortalities for randomly selected text inputs were calculated and listed.

The study was implemented using Python 3.9, Scikit-learn 0.24 [31], imbalanced-learn 0.8.0 [23], PyTorch 1.8 [32], and transformers 4.9 (Hugging Face) [24]. Our models were trained and validated on the NVIDIA Tesla P100-PCIE-16GB graphics processing unit (GPU). The statistical significances of AUROCs and AUPRCs were calculated using MedCalc software (Ostend, Belgium).

## Results

### Comparison of Machine Learning Models

**Figure 3:**
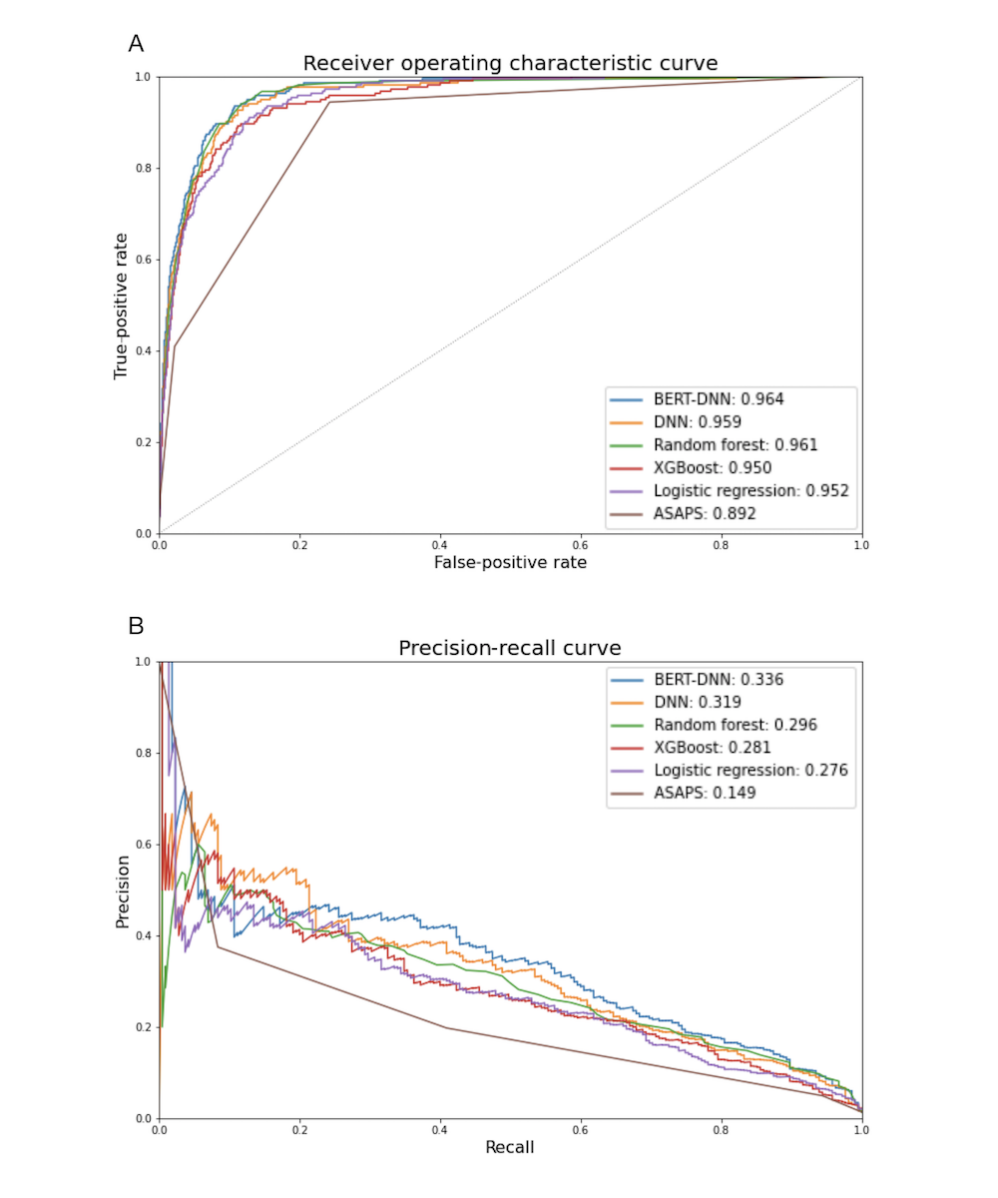
Comparison of discrimination of different models. (A) AUROC. (B) AUPRC. ASAPS: American Society of Anesthesiologist Physical Status; AUPRC: area under the precision-recall curve; AUROC: area under the receiver operating characteristic curve; BERT: bidirectional encoder representations from transformers; DNN: deep neural network; XGBoost: extreme gradient boosting.

The BERT-DNN had the highest AUROC of 0.964 (95% CI 0.961-0.967) and the highest AUPRC of 0.336 (95% CI 0.276-0.402); see Table 3 and Figure 3. The random forest achieved the second-highest AUROC of 0.961 (95% CI 0.958-0.964), and the DNN achieved the second-highest AUPRC of 0.319 (95% CI 0.260-0.384). The BERT-DNN model had the highest F1 score of 0.347 (95% CI 0.305-0.388).

The BERT-DNN had a significantly higher AUROC compared to XGBoost, logistic regression, and ASAPS but not a significantly higher AUROC compared to the DNN and the random forest (Table 4). The BERT-DNN also had a significantly higher AUPRC compared to the DNN, random forest, XGBoost, logistic regression, and ASAPS (Table 5).

**Figure 4:**
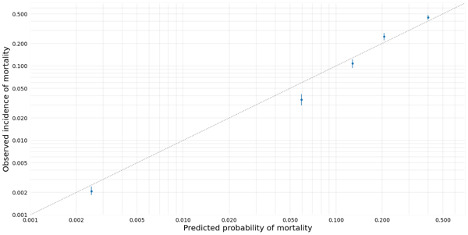
Calibration plot. The observed incidence of mortality was plotted against the calibrated predicted probability of mortality among patients in the test cohort (n=16,267, 14.1%). Predicted probabilities were calibrated by applying the histogram binning technique in the validation cohort using 5 bins. Mean predicted probabilities of in-hospital 30-day mortality were calculated within each group.

In the BERT-DNN model, when the predicted probability of mortality increased from 0.2% to 39.4%, the observed incidence increased from 0.2% to 42.7% (Figure 4).

**Table 3:** Prediction performances of ML^a^ models and ASAPS^b^ on the testing cohort with 95% CIs.

Model	AUROC^c^ (95% CI)	AUPRC^d^ (95% CI)	Accuracy^e^ (95% CI)	Sensitivity^e^ (95% CI)	Specificity^e^ (95% CI)	Precision^a^ (95% CI)	F1 score^e^ (95% CI)
BERT^f^-DNN^g^	0.964 (0.961-0.967)	0.336 (0.276-0.402)	0.955 (0.952-0.958)	0.749 (0.689-0.805)	0.958 (0.955-0.961)	0.193 (0.166-0.219)	0.307 (0.269-0.342)
DNN	0.959 (0.956-0.962)	0.319 (0.260-0.384)	0.913 (0.909-0.917)	0.885 (0.841-0.926)	0.913 (0.909-0.918)	0.120 (0.104-0.136)	0.212 (0.187-0.236)
Random forest	0.961 (0.958-0.964)	0.296 (0.239-0.360)	0.986 (0.984-0.988)	0.167 (0.122-0.222)	0.997 (0.996-0.998)	0.445 (0.341-0.557)	0.242 (0.182-0.314)
XGBoost^h^	0.950 (0.946-0.953)	0.281 (0.225-0.345)	0.986 (0.984-0.987)	0.195 (0.144-0.249)	0.996 (0.995-0.997)	0.409 (0.312-0.500)	0.263 (0.201-0.326)
Logistic regression	0.952 (0.949-0.955)	0.276 (0.220-0.339)	0.904 (0.900-0.909)	0.833 (0.780-0.882)	0.905 (0.901-0.910)	0.105 (0.091-0.119)	0.187 (0.164-0.210)
ASAPS	0.892 (0.887-0.896)	0.149 (0.107-0.203)	0.970 (0.968-0.973)	0.409 (0.342-0.478)	0.978 (0.975-0.980)	0.197 (0.160-0.235)	0.266 (0.220-0.310)

^a^ML: machine learning.

^b^ASAPS: American Society of Anesthesiologist Physical Status.

^c^AUROC: area under the receiver operating characteristic.

^d^AUPRC: area under the precision-recall curve.

^e^These metrics were calculated without adjusting the threshold (using 0.5 as the cut-off).

^f^BERT: bidirectional encoder representations from transformers.

^g^DNN: deep neural network.

^h^XGBoost: extreme gradient boosting.

**Table 4:** Statistical significances of AUROCs^a^ of different models. Values are *P* values. We applied a nonparametric approach proposed by DeLong et al [27] to calculate the SE of the area and the *P* value.

	BERT^b^-DNN^c^	DNN	Random forest	XGBoost^d^	Logistic regression
ASAPS^e^	<0.0001^f^	<0.0001^f^	<0.0001^f^	<0.0001^f^	<0.0001^f^
Logistic regression	0.0005^f^	0.0711	0.0351^f^	0.6451	N/A^g^
XGBoost	0.0025^f^	0.0939	0.0262^f^	N/A	N/A
Random forest	0.3816	0.5972	N/A	N/A	N/A
DNN	0.0944	N/A	N/A	N/A	N/A

^a^AUROC: area under the receiver operating characteristic.

^b^BERT: bidirectional encoder representations from transformers.

^c^DNN: deep neural network.

^d^XGBoost: extreme gradient boosting.

^e^ASAPS: American Society of Anesthesiologist Physical Status.

^f^The difference in areas achieved statistical significance (*P*<.05).

^g^N/A: not applicable.

**Table 5:** Statistical significances of AUPRCs^a^ of different models. Values are differences in areas with 95% CIs calculated by bootstrapping 1000 times [28]. If the 95% CI for the difference in areas does not include 0, it can be concluded that these 2 areas are significantly different (*P*<.05).

	BERT^b^-DNN^c^, difference in areas (95% CI)	DNN, difference in areas (95% CI)	Random forest, difference in areas (95% CI)	XGBoost^d^, difference in areas (95% CI)	Logistic regression, difference in areas (95% CI)
ASAPS^e^	0.188 (0.159-0.221)^f^	0.170 (0.137-0.201)^f^	0.147 (0.122-0.177)^f^	0.133 (0.107-0.162)^f^	0.127 (0.101-0.154)^f^
Logistic regression	0.061 (0.051-0.073)^f^	0.043 (0.021-0.056)^f^	0.020 (0.006-0.031)^f^	0.006 (–0.006 to 0.014)	N/A^g^
XGBoost	0.055 (0.044-0.068)^f^	0.038 (0.024-0.046)^f^	0.015 (0.005-0.022)^f^	N/A	N/A
Random forest	0.040 (0.030-0.054)^f^	0.023 (0.010-0.032)^f^	N/A	N/A	N/A
DNN	0.018 (0.008-0.037)^f^	N/A	N/A	N/A	N/A

^a^AUPRC: area under the precision-recall curve.

^b^BERT: bidirectional encoder representations from transformers.

^c^DNN: deep neural network.

^d^XGBoost: extreme gradient boosting.

^e^ASAPS: American Society of Anesthesiologist Physical Status.

^f^The difference in areas achieved statistical significance (*P*<.05).

^g^N/A: not applicable.

### Visualization of Word Embeddings

**Figure 5:**
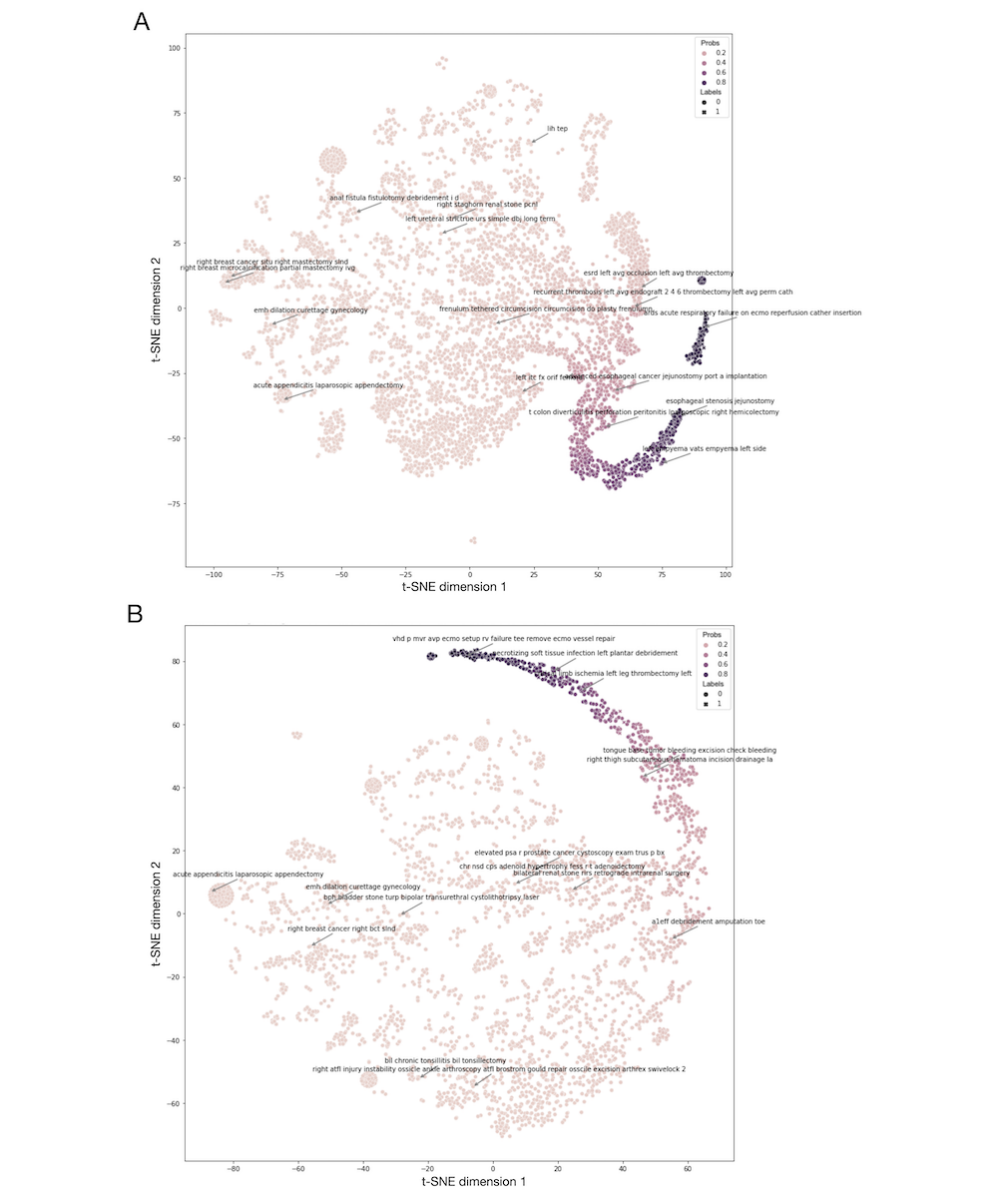
Word embeddings visualized by t distributed stochastic neighbor embedding. (A) Word embeddings of the training set. (B) Word embeddings of the testing set. “Probs” indicates probabilities predicated by the BERT-DNN model. The intensity of color increased with the probability. “Labels” indicates mortalities by “x” and survivors by “•”. ards: acute respiratory distress syndrome; atfl: anterior talofibular ligament; avg: arteriovenous graft; avp: aortic valvuloplasty; BERT: bidirectional encoder representations from transformers; bct: breast-conserving therapy; bil: bilateral; bph: benign prostate hypertrophy; bx: biopsy; chr: chronic hypertrophic rhinitis; cps: chronic paranasal sinusitis; dbj: double J stent; DNN: deep neural network; ecmo: extracorporeal membrane oxygenation; emh: endometrial hemorrhage; esrd: end-stage renal disease; fess: functional endoscopic sinus surgery; itc: intertrochanter; ivg: intravenous general anesthesia; lih: left inguinal hernia; mvr: mitral valve replacement; nsd: nasal septum deviation; p: post; pcnl: percutaneous nephrolithotomy; perm cath: permanent catheter; psa: prostate-specific antigen; r: rule out; r’t: right; rirs: retrograde intrarenal surgery; rv: right ventricle; slnd: sentinel lymph node dissection; SNE: stochastic neighbor embedding; t colon: transverse colon; tee: transesophageal echocardiography; tep: total extraperitoneal approach; trus: transrectal ultrasound; turp: transurethral resection of the prostate; urs: ureteroscopy; vats: video-assisted thoracic surgery; vhd: valvular heart disease. Higher-resolution version of this figure available in Multimedia Appendix 3.

Because the observed mortalities were distributed concordantly with increased prediction probabilities, the annotated scatter plots showed that the text contributed to low- and high-probability predictions (Figure 5, Multimedia Appendix 3). Table 6 lists the probabilities predicted by the language model and the mortalities observed for a randomly selected text input.

**Table 6:** Texts and their predicted probabilities by language model. Values are probabilities or mortalities.

Predicted probability	Observed mortality (1=mortality; 0=no mortality)	Original free text combining preoperative diagnosis and proposed procedures
0.951	1	IHCA^a^ p^b^ CPR^c^ ECMO^d^ ACS^e^ AR^f^ full sternotomy CABG^g^ AVR^h^
0.948	0	AMI^i^ cardiogenic shock p ECMO remove ECMO TEE^j^
0.940	1	hollow organ perforation r^k^ PPU^l^ related LPPU^m^ possible EXP LAP^n^
0.936	1	intra-abdominal bleeding EXP LAP
0.932	1	ischemic bowel laparoscopic diagnosis possible EXP LAP
0.927	0	acute pulmonary embolism IHCA p ECMO angiography TEE
0.925	1	duodenal ulcer perforation p duodenorrhaphy leakage bleeding EXP LAP
0.912	0	respiratory failure tracheostomy
0.880	0	hallow organ perforation r PPU LPPU
0.815	0	acute kidney failure perm cath^o^ insertion
0.760	0	post UPPP^p^ wound bleeding check bleeding
0.680	0	ESRD^q^ HD^r^ via right perm cath^s^ qw2 4 6 perm cath dysfunction perm cath insertion change perm cath right neck
0.527	0	ESRD left AVG^t^ occlusion left AVG thrombectomy
0.415	0	left lower leg soft tissue infection suspect necrotizing fasciitis debridement
0.353	0	ESRD right AVF^u^ dysfunction upper arm angiography PTA^v^
0.250	0	RLL^w^ lung tumor r lung cancer vats RLL lobectomy wedge first send frozen exam
0.186	0	left lower extremity NF^x^ open BK^y^
0.114	0	left anterior mediastinal tumor multiple lung nodules rectal cancer p CCRT^z^ VATS^aa^ mediastinal tumor excision LAR^ab^
0.042	0	right ACL^ac^ MCL^ad^ injury arthroscopy ACL reconstruction
0.041	0	1 C4 5 6 spondylosis 2 right carpal tunnel 1 ACDF^ae^ C4 5 6 2 right median nerve decompression
0.031	0	bil^af^ ov^ag^ teratoma laparoscopy adnexectomy
0.030	0	left ureter stone URSL^ah^ laser left
0.029	0	uterine myoma robotic myomectomy
0.029	0	acute appendicitis laparoscopic appendectomy
0.029	0	hemorrhoids hemorrhoidectomy
0.029	0	nontoxic goiter thyroidectomy
0.027	0	infertility TVOR^ai^
0.027	0	endometrial polyp TCR^aj^
0.027	0	GA^ak^ 38 weeks breech caesarean section
0.025	0	rt^al^ breast lesion MRI^am^ guided biopsy
0.025	0	right inguinal hernia TEP^an^ right

^a^IHCA: intrahospital cardiac arrest.

^b^p: post.

^c^CPR: cardiopulmonary resuscitation.

^d^ECMO: extracorporeal membrane oxygenation.

^e^ACS: acute coronary syndrome.

^f^AR: aortic regurgitation.

^g^CABG: coronary artery bypass graft.

^h^AVR: aortic valve replacement.

^i^AMI: acute myocardial infarction.

^j^TEE: transesophageal echocardiography.

^k^r: rule out.

^l^PPU: perforated peptic ulcer.

^m^LPPU: laparoscopic perforated peptic ulcer surgery.

^n^EXP LAP: exploratory laparotomy.

^o^cath: catheter.

^p^UPPP: uvulopalatopharyngoplasty.

^q^ESRD, end-stage renal disease.

^r^HD: hemodialysis.

^s^perm cath: permanent catheter.

^t^AVG: arteriovenous graft.

^u^AVF: arteriovenous fistula.

^v^PTA: percutaneous transluminal angioplasty.

^w^RLL: right lower lobe.

^x^NF: necrotizing fasciitis.

^y^BK: below-knee amputation.

^z^CCRT: concurrent chemoradiotherapy.

^aa^VATS: video-assisted thoracic surgery.

^ab^LAR: low anterior resection.

^ac^ACL: anterior cruciate ligament.

^ad^MCL: medial collateral ligament.

^ae^ACDF: anterior cervical discectomy and fusion.

^af^bil: bilateral.

^ag^ov: ovarian.

^ah^URSL: ureteroscopic lithotomy.

^ai^TVOR: transvaginal oocyte retrieval.

^aj^TCR: transcervical resectoscope.

^ak^GA: gestational age.

^al^rt: right.

^am^MRI: magnetic resonance imaging.

^an^TEP: total extraperitoneal approach.

## Discussion

### Principal Findings

The DNN-BERT model predicted the in-hospital 30-day mortality with the highest AUROC of 0.964 (95% CI 0.961-0.967) and an AUPRC of 0.336 (95% CI 0.276-0.402); see Table 3 and Figure 3. The BERT-DNN had an AUROC significantly higher compared to XGBoost, logistic regression, and ASAPS but not the DNN or random forest. The BERT-DNN also had an AUPRC significantly higher compared to the DNN, random forest, XGBoost, logistic regression, and ASAPS.

Hill et al [6] proposed an ML model that outperformed previous tools (eg, preoperative score to predict postoperative mortality, Charlson comorbidity, and ASAPS) and could be used independently by clinicians. Our BERT-DNN model outperformed Hill et al’s [6] model, obtaining a higher AUROC, sensitivity, and F1 score than their results (0.964, 95% CI 0.961-0.967 vs 0.932, 95% CI 0.910-0.951; 0.650, 95% CI 0.587-0.719 vs 0.239, 95% CI 0.127-0.379; and 0.347, 95% CI 0.305-0.388 vs 0.302, 95% CI 0.172-0.449, respectively); see Table 3. The preoperative diagnosis text features and proposed procedure information might contribute to our BERT-DNN model and enhance its sensitivity and F1 score. Unlike Hill et al [6], who focused on patients undergoing general anesthesia, we trained and tested our model on both general and neuraxial anesthesia. The DL model with clinical text predicted postoperative mortality significantly more discriminatively than logistic regression and ASAPS (Table 4).

DL methods predict postoperative mortality using preoperative and intraoperative features [7-9]. Using a summary of intraoperative features alongside the ASAPS, Lee et al [7] presented a DNN model that achieved an AUROC of 0.91 (95% CI 0.88-0.93). Our DNN model obtained a higher AUROC than their model because we included key features such as preoperative location and surgical department, the importance of which was also verified in previous studies [6]. Fritz et al [8] proposed a multipath convolutional neural network model to predict postoperative mortality using intraoperative time-series data and preoperative features. Their model achieved an AUROC of 0.910 (95% CI 0.897-0.924) and an AUPRC of 0.325 (95% CI 0.280-0.372) [33]. In contrast, our model can be used preoperatively and achieve a higher AUROC and AUPRC (Table 3).

Previous studies used *ICD* and CPT codes as categorical features to stratify surgery risk [2,6,7,9,12]. This input feature has many classes, which resulted in a sparse input matrix; this made it difficult for the model to learn helpful information. However, because *ICD* codes were typically recorded after surgery, including them in the preoperative model was impractical. Furthermore, the CPT code was not used globally. For this reason, we could not compare a model including word embeddings with one including CPT codes. However, our results exhibited excellent discrimination with a high AUROC and AUPRC. The AUPRC is significantly higher than models without text. The calibration plot also strongly correlated the predicted probabilities and observed mortalities (Figure 4). Word embedding visualizations showed that the increased predicted probabilities were concordant with high-risk surgery and an increased mortality rate (Figure 5 and Table 6). We showed that word embeddings for surgery information could be used in DL models to predict postoperative mortality before surgery without requiring CPT or *ICD* codes.

The fusion of neural networks, combining diverse types of data (eg, image [34] and time-series [8] data) with 1D data (eg, categorical, and continuous data), improved the model’s performance. Including unstructured clinical text via natural language procession can improve intensive care unit (ICU) mortality predictions [14,16]. The DL model that combined unstructured and structured data outperformed models using either type of data alone [15]. Moreover, the performance of the clinical pretrained DL language model could be maintained between different institutions [35].

### Limitations

Our study has several limitations. First, postoperative mortality accounted for 1.3% (1562/121,313) of our cohort, and the classes were highly imbalanced. The model training and performance metric evaluations were difficult to apply with these sparse positive labels. To compensate for the class imbalance via an algorithmic method, we applied cost-sensitive learning by balancing the weights of the loss function to emphasize the minority group [36]. We evaluated the discrimination of our model with the AUPRC, which is more informative than the AUROC for imbalanced data [8,25,26]. Second, our model predicted mortality using EHRs. The errors in the records and missing values affected the prediction results. Typos of text interfered with the word-embedding process. Outliers were detected and input using the defined rules (Multimedia Appendix 2). Third, all records were collected from a single large medical center. Although the pipeline we created ensured that the DL model could be reproduced in other institutes, the model weights might vary for a different data set. The generalizability of our results must be examined in future studies.

### Conclusion

In conclusion, descriptive surgical text was essential for predicting postoperative mortality. The word embeddings of preoperative diagnoses and proposed procedures, via the contextualized language model BERT, were combined in DL models to predict postoperative mortality. This predictive capacity can help identify patients with higher risk from structure data and text of EHRs.

## Appendix

Multimedia Appendix 1 Summary of laboratory values and vital sign values.

Multimedia Appendix 2 Continuous feature limits to define outliers.

Multimedia Appendix 3 Higher resolution of Figure 5. Word embeddings visualized by t distributed stochastic neighbor embedding. (A) Word embeddings of the training set. (B) Word embeddings of the testing set. “Probs” indicates probabilities predicated by the BERT-DNN model. The intensity of color increased with the probability. “Labels” indicates mortalities by “x” and survivors by “•”. ards: acute respiratory distress syndrome; atfl: anterior talofibular ligament; avg: arteriovenous graft; avp: aortic valvuloplasty; BERT: bidirectional encoder representations from transformers; bct: breast-conserving therapy; bil: bilateral; bph: benign prostate hypertrophy; bx: biopsy; chr: chronic hypertrophic rhinitis; cps: chronic paranasal sinusitis; dbj: double J stent; DNN: deep neural network; ecmo: extracorporeal membrane oxygenation; emh: endometrial hemorrhage; esrd: end-stage renal disease; fess: functional endoscopic sinus surgery; itc: intertrochanter; ivg: intravenous general anesthesia; lih: left inguinal hernia; mvr: mitral valve replacement; nsd: nasal septum deviation; p: post; pcnl: percutaneous nephrolithotomy; perm cath: permanent catheter; psa: prostate-specific antigen; r: rule out; r’t: right; rirs: retrograde intrarenal surgery; rv: right ventricle; slnd: sentinel lymph node dissection; SNE: stochastic neighbor embedding; t colon: transverse colon; tee: transesophageal echocardiography; tep: total extraperitoneal approach; trus: transrectal ultrasound; turp: transurethral resection of the prostate; urs: ureteroscopy; vats: video-assisted thoracic surgery; vhd: valvular heart disease.

## Abbreviations

ASAPS: American Society of Anesthesiologist Physical Status
AUPRC: area under the precision-recall curve
AUROC: area under the receiver operator characteristic curve
BERT: bidirectional encoder representations from transformers
CPT: Current Procedural Terminology
DL: deep learning
DNN: deep neural network
EHR: electronic health record
FC: fully connected
ICD: International Classification of Diseases
ML: machine learning
XGBoost: extreme gradient boosting

## References

[1] International Surgical Outcomes Study Group (2016). Global patient outcomes after elective surgery: prospective cohort study in 27 low-, middle- and high-income countries. Br J Anaesth.

[2] Sigakis M, Bittner E, Wanderer J (2013). Validation of a risk stratification index and risk quantification index for predicting patient outcomes: in-hospital mortality, 30-day mortality, 1-year mortality, and length-of-stay. Anesthesiology.

[3] Le Manach Y, Collins G, Rodseth R, Le Bihan-Benjamin C, Biccard B, Riou B, Devereaux PJ, Landais P (2016). Preoperative Score to Predict Postoperative Mortality (POSPOM): derivation and validation. Anesthesiology.

[4] Mayhew D, Mendonca V, Murthy BVS (2019). A review of ASA physical status: historical perspectives and modern developments. Anaesthesia.

[5] Bilimoria K, Liu Y, Paruch J, Zhou L, Kmiecik T, Ko C, Cohen ME (2013). Development and evaluation of the universal ACS NSQIP surgical risk calculator: a decision aid and informed consent tool for patients and surgeons. J Am Coll Surg.

[6] Hill BL, Brown R, Gabel E, Rakocz N, Lee C, Cannesson M, Baldi P, Olde Loohuis L, Johnson R, Jew B, Maoz U, Mahajan A, Sankararaman S, Hofer I, Halperin E (2019). An automated machine learning-based model predicts postoperative mortality using readily-extractable preoperative electronic health record data. Br J Anaesth.

[7] Lee CK, Hofer I, Gabel E, Baldi P, Cannesson M (2018). Development and validation of a deep neural network model for prediction of postoperative in-hospital mortality. Anesthesiology.

[8] Fritz BA, Cui Z, Zhang M, He Y, Chen Y, Kronzer A, Ben Abdallah A, King CR, Avidan MS (2019). Deep-learning model for predicting 30-day postoperative mortality. Br J Anaesth.

[9] Yan X, Goldsmith J, Mohan S, Turnbull Z, Freundlich R, Billings F, Kiran RP, Li G, Kim M (2022). Impact of intraoperative data on risk prediction for mortality after intra-abdominal surgery. Anesth Analg.

[10] Konishi T, Goto T, Fujiogi M, Michihata N, Kumazawa R, Matsui H, Fushimi K, Tanabe M, Seto Y, Yasunaga H (2022). New machine learning scoring system for predicting postoperative mortality in gastroduodenal ulcer perforation: a study using a Japanese nationwide inpatient database. Surgery.

[11] Rogers MP, Janjua H, DeSantis AJ, Grimsley E, Pietrobon R, Kuo PC (2022). Machine learning refinement of the NSQIP risk calculator: who survives the "Hail Mary" case?. J Am Coll Surg.

[12] Dalton J, Kurz A, Turan A, Mascha E, Sessler D, Saager L (2011). Development and validation of a risk quantification index for 30-day postoperative mortality and morbidity in noncardiac surgical patients. Anesthesiology.

[13] Hashimoto D, Witkowski E, Gao L, Meireles O, Rosman G (2020). Artificial intelligence in anesthesiology: current techniques, clinical applications, and limitations. Anesthesiology.

[14] Weissman GE, Hubbard RA, Ungar LH, Harhay MO, Greene CS, Himes BE, Halpern SD (2018). Inclusion of unstructured clinical text improves early prediction of death or prolonged ICU stay. Crit Care Med.

[15] Zhang D, Yin C, Zeng J, Yuan X, Zhang P (2020). Combining structured and unstructured data for predictive models: a deep learning approach. BMC Med Inform Decis Mak.

[16] Marafino BJ, Park M, Davies JM, Thombley R, Luft HS, Sing DC, Kazi DS, DeJong C, Boscardin WJ, Dean ML, Dudley RA (2018). Validation of prediction models for critical care outcomes using natural language processing of electronic health record data. JAMA Netw Open.

[17] Devlin J, Chang M, Lee K, Toutanova K (2018). Bert: Pre-training of deep bidirectional transformers for language understanding. arXiv preprint.

[18] Elbattah M, Gignon M, Dequen G, Arnaud (2022). Learning embeddings from free-text triage notes using pretrained transformer models.

[19] Kades K, Sellner J, Koehler G, Full PM, Lai TYE, Kleesiek J, Maier-Hein KH (2021). Adapting bidirectional encoder representations from transformers (BERT) to assess clinical semantic textual similarity: algorithm development and validation study. JMIR Med Inform.

[20] Loper E, Bird S (2002). Nltk: The natural language toolkit. arXiv.

[21] Breiman L (2001). Random forests. Mach Learn.

[22] Chen T, Guestrin C (2016). Xgboost: a scalable tree boosting system.

[23] Chawla NV, Bowyer KW, Hall LO, Kegelmeyer WP (2002). SMOTE: Synthetic Minority Over-sampling Technique. J Artif Intell Res.

[24] Alsentzer E, Murphy J, Boag W, Weng W, Jin D, Naumann T, McDermott M (2019). Publicly available clinical BERT embeddings. arXiv.

[25] Saito T, Rehmsmeier M (2015). The precision-recall plot is more informative than the ROC plot when evaluating binary classifiers on imbalanced datasets. PLoS One.

[26] Ozenne B, Subtil F, Maucort-Boulch D (2015). The precision-recall curve overcame the optimism of the receiver operating characteristic curve in rare diseases. J Clin Epidemiol.

[27] DeLong E, DeLong D, Clarke-Pearson D (1988). Comparing the areas under two or more correlated receiver operating characteristic curves: a nonparametric approach. Biometrics.

[28] Boyd K, Eng K, Page C, Blockeel H, Kersting K, Nijssen S, Železný F (2013). Area under the precision-recall curve: point estimates and confidence intervals. Machine Learning and Knowledge Discovery in Databases.

[29] Maaten LVD, Hinton G (2008). Visualizing data using t-SNE. J Mach Learn Res.

[30] Jin M, Bahadori M, Colak A, Bhatia P, Celikkaya B, Bhakta R, Senthivel S, Khalilia M, Navarro D, Zhang B (2018). Improving hospital mortality prediction with medical named entities and multimodal learning. arXiv.

[31] Pedregosa F, Varoquaux G, Gramfort A, Michel V, Kossaifi J, Thirion B, Grisel O, Blondel M, Prettenhofer P, Weiss R, Dubourg V, Vanderplas J, Passos A, Cournapeau D, Brucher M, Perrot M, Duchesnay É (2011). Scikit-learn: machine learning in Python. J Mach Learn Res.

[32] Paszke A, Gross S, Massa F, Lerer A, Bradbury J, Chanan G, Killeen T, Lin Z, Gimelshein N, Antiga L (2019). Pytorch: an imperative style, high-performance deep learning library.

[33] Fritz BA, Abdelhack M, King CR, Chen Y, Avidan MS (2020). Update to 'Deep-learning model for predicting 30-day postoperative mortality' (Br J Anaesth 2019; 123: 688-95). Br J Anaesth.

[34] Huang S, Pareek A, Seyyedi S, Banerjee I, Lungren MP (2020). Fusion of medical imaging and electronic health records using deep learning: a systematic review and implementation guidelines. NPJ Digit Med.

[35] Bear Don't Walk Iv OJ, Sun T, Perotte A, Elhadad N (2021). Clinically relevant pretraining is all you need. J Am Med Inform Assoc.

[36] Johnson JM, Khoshgoftaar TM (2019). Survey on deep learning with class imbalance. J Big Data.

