# Supplementary material for: Predicting Postoperative Mortality With Deep Neural Networks and Natural Language Processing: Model Development and Validation
Source: JMIR Med Inform. 2022 May 10;10(5):e38241. doi: 10.2196/38241 (PMC9131148; doi:10.2196/38241)
Supplement: Multimedia Appendix 1 [file medinform_v10i5e38241_app1.doc]

**Summary of laboratory values. Values are medians (25th, 75th percentile). Testing cohort was split by time between training and validation cohort and those cases arising before the 30-day period had elapsed, to prevent data leakage. (n=5,890)**

| **Feature** | **Training cohort** | **Validation cohort** | **Testing cohort** | **Overall cohort** |
| --- | --- | --- | --- | --- |
|  | n = 79,324 | n = 19,832 | n = 16,267 | n = 121,313 |
| Hemoglobin (g/dL) | 13.2 (11.90, 14.40) | 13.2 (11.90, 14.30) | 13.2 (12.00, 14.60) | 13.2 (11.90, 14.40) |
| Hematocrit (%) | 39.7 (36.60, 42.30) | 39.7 (36.50, 42.20) | 39.7 (36.90, 42.90) | 39.7 (36.60, 42.30) |
| Platelet (10^3^/μL) | 242.0 (206.00, 281.00) | 242.0 (207.00, 280.00) | 242.0 (206.00, 289.00) | 242.0 (206.00, 281.00) |
| Prothrombin time (sec) | 1.0 (0.98, 1.03) | 1.0 (0.98, 1.03) | 1.0 (0.98, 1.03) | 1.0 (0.98, 1.03) |
| International normalized ratio | 10.2 (10.00, 10.50) | 10.2 (10.00, 10.40) | 10.2 (10.00, 10.40) | 10.2 (10.00, 10.40) |
| Activated partial thromboplastin time (sec) | 27.3 (26.40, 28.50) | 27.3 (26.40, 28.50) | 27.3 (25.90, 28.35) | 27.3 (26.40, 28.50) |
| Blood urea nitrogen (mg/dL) | 15.0 (15.00, 15.00) | 15.0 (15.00, 15.00) | 15.0 (15.00, 15.00) | 15.0 (15.00, 15.00) |
| Creatinine (mg/dL) | 0.75 (0.63, 0.89) | 0.75 (0.63, 0.89) | 0.75 (0.60, 0.88) | 0.75 (0.63, 0.90) |
| Aspartate transaminase (Unit/L) | 21.0 (20.00, 21.00) | 21.0 (20.00, 21.00) | 21.0 (21.00, 21.00) | 21.0 (20.00, 21.00) |
| Alanine transaminase (Unit/L) | 19.0 (17.00, 21.00) | 19.0 (17.00, 21.00) | 19.0 (15.00, 23.00) | 19.0 (16.00, 21.00) |
| Blood sugar (mg/dL) | 111.0 (102.00, 122.00) | 111.0 (102.00, 123.00) | 111.0 (102.00, 124.00) | 111.0 (102.00, 123.00) |
| Sodium (mmol/L) | 139.0 (138.00, 141.00) | 139.0 (138.00, 141.00) | 139.0 (137.00, 140.00) | 139.0 (138.00, 141.00) |
| Potassium (mmol/L) | 4.0 (3.80, 4.10) | 4.0 (3.80, 4.10) | 4.0 (3.80, 4.10) | 4.0 (3.80, 4.10) |

**Summary of vital sign values.** Values are medians (25th, 75th percentile). Testing cohort was split by time between training and validation cohort and those cases arising before 30-day period had elapsed, to prevent data leakage. (n=5,890)

| **Feature** | **Training cohort** | **Validation cohort** | **Testing cohort** | **Overall cohort** |
| --- | --- | --- | --- | --- |
|  | n = 79,324 | n = 19,832 | n = 16,267 | n = 121,313 |
| Body temperature (C^o^) | 36.5 (36.30, 36.70) | 36.5 (36.30, 36.70) | 36.5 (36.10, 36.90) | 36.5 (36.20, 36.70) |
| Pulse oximeter (%) | 97.0 (97.00, 98.00) | 97.0 (97.00, 98.00) | 97.0 (96.00, 98.00) | 97.0 (97.00, 98.00) |
| Heart rate (min^-1^) | 77.0 (72.00, 82.00) | 77.0 (73.00, 82.00) | 77.0 (69.00, 88.00) | 77.0 (72.00, 83.00) |
| Respiratory rate (min^-1^) | 14.0 (14.00, 15.00) | 14.0 (14.00, 15.00) | 14.0 (14.00, 15.00) | 14.0 (14.00, 15.00) |
| Systolic blood pressure (mmHg) | 146.0 (137.00, 156.00) | 146.0 (136.00, 155.00) | 146.0 (128.00, 161.00) | 146.0 (135.00, 157.00) |
| Diastolic blood pressure (mmHg) | 80.0 (76.00, 85.00) | 80.0 (76.00, 84.00) | 80.0 (72.00, 88.00) | 80.0 (75.00, 85.00) |
