# Supplementary material for: Predicting Postoperative Mortality With Deep Neural Networks and Natural Language Processing: Model Development and Validation
Source: JMIR Med Inform. 2022 May 10;10(5):e38241. doi: 10.2196/38241 (PMC9131148; doi:10.2196/38241)
Supplement: Multimedia Appendix 2 [file medinform_v10i5e38241_app2.doc]

**Continuous feature limits to define outliers**

| **Feature** | **Lower limit** | **Upper limit** |
| --- | --- | --- |
| Body weight (kg) | 25 | 200 |
| Body height (cm) | 120 | 250 |
| Body mass index (kg · m^-2^) |  | 100 |
| Body temperature (C^o^) | 32 | 42 |
| Pulse oximeter (%) | 0 | 100 |
| Heart rate (min^-1^) | 0 | 250 |
| Respiratory rate (min^-1^) | 0 | 35 |
| Systolic blood pressure (mmHg) | 0 | 300 |
| Diastolic blood pressure (mmHg) | 0 | 300 |
| Hemoglobin (g/dL) |  | 25 |
| Hematocrit (%) |  | 75 |
