# Supplementary figures and images for: Predicting Postoperative Mortality With Deep Neural Networks and Natural Language Processing: Model Development and Validation
Source: JMIR Med Inform. 2022 May 10;10(5):e38241. doi: 10.2196/38241 (PMC9131148; doi:10.2196/38241)

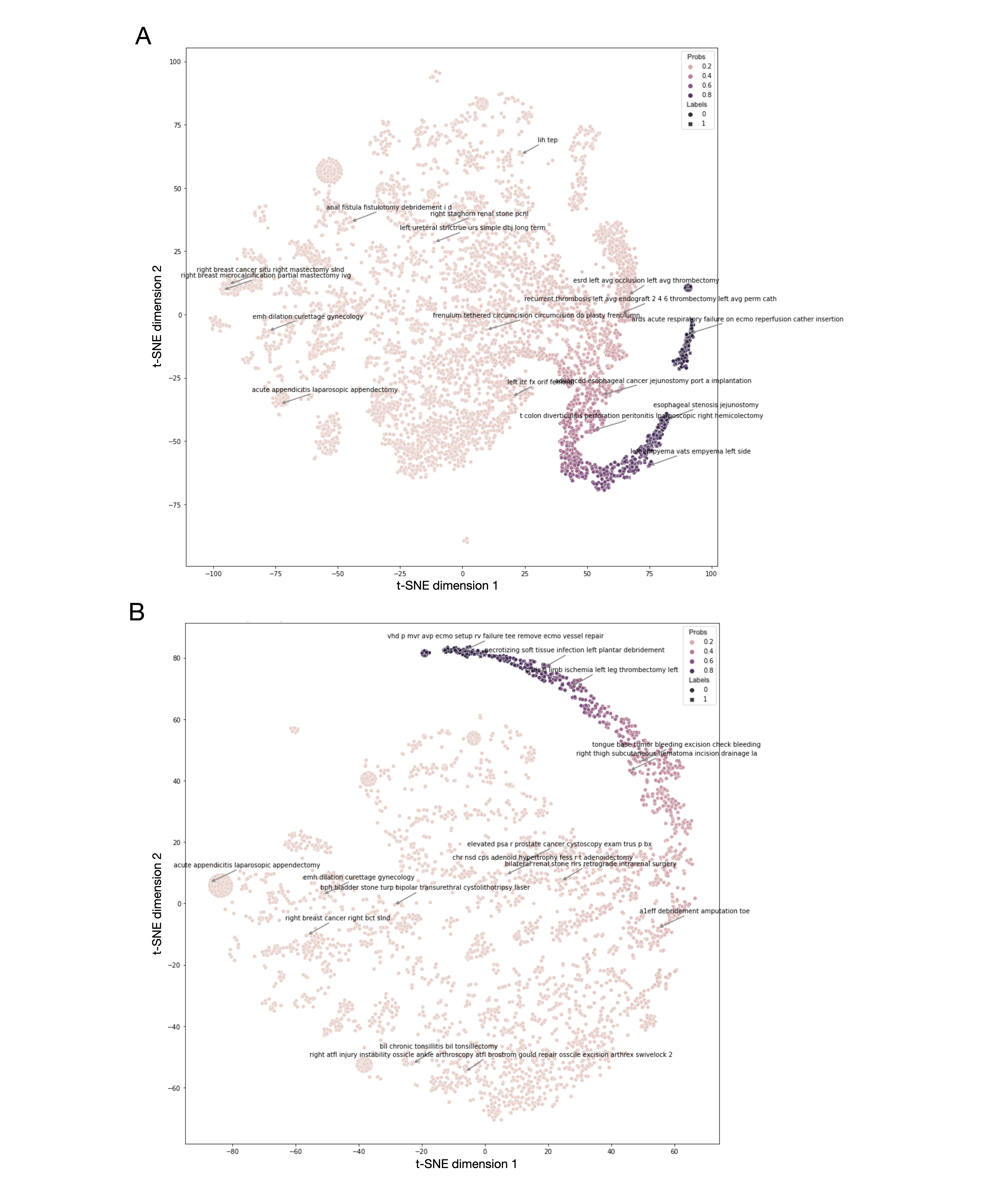

Supplement: Multimedia Appendix 3 [file medinform_v10i5e38241_app3.png]
